# Lipoprotein-associated phospholipase A2 and oxidized low-density lipoprotein in young patients with acute coronary syndrome in China

**DOI:** 10.1038/s41598-017-16464-5

**Published:** 2017-11-23

**Authors:** Yuli Huang, Yu Wu, You Yang, Wensheng Li, Jianhua Lu, Yunzhao Hu

**Affiliations:** Department of Cardiology, Shunde Hospital, Southern Medical University, Foshan, China

## Abstract

Lipoprotein-associated phospholipase A2 (Lp-PLA2) is considered to be a risk factor for acute coronary syndrome (ACS), but this remains controversial. This study investigated the role of Lp-PLA2 in young Chinese patients with ACS. 228 young patients (aged ≤55 years) with ACS and 237 age-matched controls were included. Lp-PLA2 and oxidized low-density lipoprotein (ox-LDL) levels were measured by sandwich enzyme-linked immunosorbent assay. Lp-PLA2 levels were significantly correlated with smoking, total cholesterol (TC), low-density lipoprotein cholesterol (LDL-C) and ox-LDL levels (all *P* < 0.05). Multivariate logistic regression analysis showed that male sex (OR = 3.25, 95%CI = 1.26–8.38), smoking (OR = 3.50, 95%CI = 1.75–7.0), triglyceride (OR = 1.76, 95%CI = 1.08–2.87), high sensitivity C-reactive protein (hs-CRP) (OR = 2.11, 95%CI = 1.14–3.90) and ox-LDL (OR = 2.98, 95%CI = 1.72–5.1) were independently associated with ACS risk in young patients. Lp-PLA2 was associated with risk of ACS in young patients when adjusted for traditional risk factors, including age, sex, diabetes, hypertension, smoking, TC, LDL-C, triglyceride and hs-CRP (OR = 1.98, 95%CI = 1.10–3.56). When further adjusted for ox-LDL levels, the association between Lp-PLA2 and ACS became insignificant (OR = 1.69, 95%CI = 0.90–3.17). Lp-PLA2 was a marker of oxidative stress and inflammation, rather than an independent risk factor for ACS in young Chinese patients.

## Introduction

Coronary artery disease (CAD) is a major cause of death worldwide, including China^1,2^. Although it is generally associated with older age, CAD has recently been reported more frequently in younger individuals^3^. It has been reported that patients younger than 55 years of age account for 23% of those with acute coronary syndrome (ACS)^4^. This situation has also become common during the past decade in China, causing a significant burden on health care costs and society productiveness^5^.

The risk factors for ACS in young patients are different from those in older patients^3^. Young patients with ACS are less likely to have traditional risk factors for cardiovascular disease (CVD), such as hypertension, dyslipidaemia and diabetes mellitus (DM), than older patients. A recent study showed that in young patients (age <50 years) with myocardial infarction, 36% had none or only one traditional risk factor for ACS and would have been classified as low risk according to these traditional risk factors assessment^6^. Therefore, other novel risk factors should be evaluated in these young patients. It has been reported that plasma homocysteine, γ-glutamyl transferase, triglycerides, lipoprotein subfractions and fibrinogen may be associated with an increased risk of CAD in young patients^7–11^, and most of these may be mediated by vascular inflammation.

Lipoprotein-associated phospholipase A2 (Lp-PLA2), a unique member of the phospholipase A2 superfamily, is secreted in the active form by T lymphocytes, monocyte-derived macrophages and mast cells^12^. Lp-PLA2 has high specificity for vascular inflammation and been proposed to be a risk marker of CVD based on many epidemiological studies and meta-analyses^13,14^. However, most of these studies have not focused on young patients with ACS. Furthermore, Lp-PLA2 is primarily bound to low-density lipoprotein (LDL) in the circulation. By hydrolysing the oxidized phosphatidylcholine component of oxidized LDL (ox-LDL), Lp-PLA2 generates two potent proinflammatory and proatherogenic mediators, oxidized free fatty acids and lysophospholipids, which can cause significant inflammatory responses in the vascular wall and atherogenesis^15^. Our prior study found that ox-LDL was an important independent risk factor for ACS in young patients^16^. However, whether this association involves Lp-PLA2 was unknown.

Therefore, this study aimed to evaluate the association between Lp-PLA2 and risk of ACS after adjusting for traditional cardiovascular risk factors and further assess the correlation between Lp-PLA2 and ox-LDL in these patients.

## Results

### Demographics and clinical characteristics of patients

We screened 242 young patients diagnosed with ACS by coronary angiography (CAG) and 248 age-matched controls. We excluded 14 patients and 11 controls based on the exclusion criteria (five patients with ACS and three controls with severe renal dysfunction, three patients with ACS with severe hepatic insufficiency, five controls with a history of anxiety or depression and six patients with ACS and three controls with uncontrolled infectious diseases). Therefore, 228 young patients with ACS (172 men, 56 women) and 237 age-matched controls (112 men, 125 women) were enrolled. Of patients diagnosed with ACS, 62 were classified as unstable angina, 94 as ST-segment elevation myocardial infarction and 72 as non-ST-segment elevation myocardial infarction. The demographics and clinical characteristics of all the participants are shown in Table 1.

**Table 1:** Demographic and clinical characteristics of patients.

	ACS group (n = 228)	Control group (n = 237)
Age (years)	48.6 ± 8.5	50.2 ± 10.6
Men [n(%)]	172 (75.4%)	112 (47.3%)^#^
CVD family history [n(%)]	16 (7.0%)	12 (5.1%)
Current smokers [n(%)]	89 (39.0%)	39 (16.5%)^#^
Hypertension [n(%)]	42 (18.4%)	38 (16.0%)
Systolic blood pressure (mm Hg)	121.1 ± 11.4	123.4 ± 13.2
Diastolic blood pressure (mm Hg)	72.5 ± 7.9	73.4 ± 8.8
Diabetes mellitus [n(%)]	24 (10.5%)	22 (9.3%)
Fasting blood glucose (mmol/L)	5.2 ± 0.9	5.1 ± 0.8
Dyslipidemia [n(%)]	73 (32.0%)	58 (24.5%)*
TC (mmol/L) 0.02586	4.9 ± 1.0	4.8 ± 1.0
LDL-C (mmol/L)	3.4 ± 0.7	3.3 ± 0.7
HDL-C (mmol/L)	1.1 ± 0.3	1.1 ± 0.4
TG (mmol/L) 0.01129	2.0 ± 0.3	1.7 ± 0.3^#^
Overweight/ Obesity [n(%)]	72 (31.6%)	64 (27.0%)
Body mass index (kg/m^2^)	24.1 ± 4.1	23.7 ± 4.6
eGFR (mL/min/1.73 m2)	110.8 ± 35.2	115.4 ± 39.8
hs-CRP (mg/dl)	4.2 ± 2.4	2.3 ± 1.8^#^
Ox-LDL(mg/dl)	3.15 ± 1.89	1.21 ± 0.82^#^
Lp-PLA2 (ng/ml)	187.4 ± 65.3	132.5 ± 53.3^#^
10-year CAD event risk (%)	5.6 ± 3.4	5.0 ± 3.9

Abbreviations: ACS, acute coronary syndrome; CVD, Cardiovascular disease; eGFR, estimated glomerular filtration rate; TC, Total cholesterol; TG, Triglyceride; HDL-C, High density lipoprotein-cholesterol; LDL-C, Low density lipoprotein-cholesterol; hs-CRP, high-sensitivity C-reactive protein; Ox-LDL, oxidized low-density lipoprotein; Lp-PLA2, Lipoprotein-associated phospholipase A2. *P < 0.05, ^#^ *P* < 0.01.

Compared with the control group, the ACS group had a greater percentage of males, current smokers and patients with dyslipidaemia (all *P* < 0.05), but there were no differences in family history of CVD, hypertension, DM or overweight or obesity (all *P* > 0.05). Ox-LDL, Lp-PLA2, TG and high sensitivity C-reactive protein (hs-CRP) levels were significantly higher in patients with ACS than those in the controls (all *P* < 0.01, Table 1), but there were no differences observed in other cardiovascular risk factors between the two groups (all *P* > 0.05) (Table 1).

### Correlation between variables and risk factors for ACS in young patients

Correlations between Lp-PLA2 and other clinical variables are shown in Table 2. Lp-PLA2 levels were significantly positively correlated with smoking status (*r* = 0.31, *P* = 0.011) and total cholesterol (TC) (*r* = 0.23, *P* = 0.032), low-density lipoprotein cholesterol (LDL-C) (*r* = 0.42, *P* = 0.001) and ox-LDL (*r* = 0.52, *P* < 0.001) levels but not with age, DM, hypertension, body mass index (BMI), hs-CRP or estimated glomerular filtration rate (eGFR) (all *P* > 0.05). Hs-CRP levels were also significantly correlated with smoking (*r* = 0.28*, P* < 0.001).

**Table 2:** Correlation of Lipoprotein-associated phospholipase A2 and other cardiovascular risk factors.

Variables	r value	P value
Age	0.05	0.530
Sex	0.15	0.150
Smoking	0.31	0.011
Hypertension	0.08	0.564
DM	−0.09	0.121
TC	0.23	0.032
LDL-C	0.42	0.001
HDL-C	−0.14	0.086
TG	0.07	0.124
BMI	0.11	0.985
eGFR	0.18	0.075
hs-CRP	0.21	0.069
Ox-LDL	0.52	<0.001

Abbreviations: BMI, body mass index; DM, diabetes mellitus; eGFR, estimated glomerular filtration rate; HDL-C, High density lipoprotein-cholesterol; hs-CRP, high-sensitivity C-reactive protein; LDL-C, low density lipoprotein-cholesterol; Ox-LDL, oxidized low-density lipoprotein; TC, total cholesterol; TG, triglyceride.

Multivariate logistic regression analysis revealed that male sex (OR = 3.25, 95%CI = 1.26–8.38, *P* = 0.015), smoking (OR = 3.50, 95%CI = 1.75–7.0, *P* < 0.001), TG (OR = 1.76, 95%CI = 1.08–2.87, *P = *0.023), hs-CRP (OR = 2.11, 95%CI = 1.14–3.90, *P* = 0.017) and ox-LDL (OR = 2.98, 95%CI = 1.72–5.13, *P* < 0.001) were independently associated with risk of ACS in young patients (Table 3). Lp-PLA2 was also associated with risk of ACS in young patients when adjusted for traditional risk factors, including age, sex, DM, hypertension, smoking, TC, LDL-C, TG and hs-CRP (OR = 1.98, 95%CI = 1.10–3.56, *P* = 0.023). However, when further adjusted for ox-LDL levels, the association between Lp-PLA2 and risk of ACS became insignificant (OR = 1.69, 95%CI = 0.90–3.17, *P* = 0.103). Finally, we performed multicollinearity analysis and found that the variance inflation factor value was >2.5 and the tolerance factor was <0.4, which indicate that there was no obvious multicollinearity in the logistic regression models.

**Table 3:** Risk factors for ACS in young patients in multivariate logistic regression analysis.

Risk factors	OR	95% CI	P-value
Sex (male vs female)	3.25	1.26–8.38	0.015
Age (per 10 years)	1.37	0.97–1.93	0.074
Smoking (yes *vs* no)	3.50	1.75–7.0	<0.001
CVD family history (yes *vs* no)	1.09	0.37–3.21	0.875
BMI (≥24 kg/m^2^ *vs* <24 kg/m^2^)	1.35	0.95–1.92	0.094
DM (yes *vs* no)	1.48	0.65–3.37	0.3504
Hypertension (yes *vs* no)	1.29	0.90–1.85	0.167
TG (≥1.7 mmol/L *vs* <1.7 mmol/L)	1.76	1.08–2.87	0.023
TC (≥5.18 mmol/L *vs* <5.18 mmol/L)	1.18	0.75–1.87	0.474
LDL-C (≥3.37 mmol/L *vs* <3.37 mmol/L)	1.29	0.96–1.73	0.093
HDL-C (≥1.04 mmol/L *vs* <1.04 mmol/L)	0.89	0.32–2.48	0.823
eGFR (per 10 mL/min/1.73 m^2^)	1.07	0.76–1.51	0.698
hs-CRP (≥3 mg/dL *vs* <3 mg/dL)	2.11	1.14–3.90	0.017
Ox-LDL (per mg/dl)	2.98	1.72–5.13	<0.001
Lp-PLA2 (per 10 ng/ml)	1.69	0.90–3.17	0.103

Abbreviations: ACS, acute coronary syndrome; BMI, body mass index; CVD, Cardiovascular disease; DM, diabetes mellitus; CI, confidence interval; eGFR, estimated glomerular filtration rate; HDL-C, high-density lipoprotein cholesterol; hs-CRP, high-sensitivity C-reactive protein; LDL-C, low-density lipoprotein cholesterol; Lp-PLA2, Lipoprotein-associated phospholipase A2; OR, odds ratio; Ox-LDL, oxidized low-density lipoprotein; TC, total cholesterol; TG, triglyceride.

## Discussion

In this study, we investigated the association between Lp-PLA2 and risk of ACS in young Chinese patients. To our knowledge, this is the first study to report that Lp-PLA2 is positively associated with ox-LDL level, which has been reported to be an independent risk factor for ACS in young Chinese patients^16^. The risk of ACS in young patients was associated with elevated Lp-PLA2 after adjusting for conventional risk factors. However, when further adjusting for ox-LDL levels, the observed association between Lp-PLA2 and ACS became insignificant, indicating that the link between Lp-PLA2 and the risk of ACS may be dependent on ox-LDL levels.

Human Lp-PLA2 is encoded by the PLA2G7 gene and secreted by inflammatory cells, such as monocytes/macrophages and mast cells, in atherosclerotic plaques^17^. In the circulation, Lp-PLA2 is bound to lipoproteins, predominantly to LDL (approximately 80%) and HDL (approximately 20%)^17^. Lp-PLA2 can be measured by mass or activity for quantification. Many epidemiological studies have observed elevated Lp-PLA2 mass or activity levels to be associated with the risk of CVD. The meta-analysis by the Lp-PLA2 Studies Collaboration (including 79036 participants in 32 prospective studies) found that both Lp-PLA2 activity and mass were continuously associated with the risk of CAD and ischemic stroke after adjusting for conventional risk factors^14^. However, most of these studies were focused on the elderly, and few were performed in Asian populations. It has been observed that higher Lp-PLA2 levels in subjects with CAD are linked to ethnicity. The Multi-Ethnic Study of Atherosclerosis showed that the association between Lp-PLA2 mass and CAD was weaker in Chinese participants^18^. Furthermore, a recently published Multi-Ethnic Study in China showed that Lp-PLA2 levels were positively associated with CAD severity. However, there was a clear positive interaction between Lp-PLA2 and classical risk factors in predicting CAD, especially for age. The proportion of CAD attributable to the interaction between Lp-PLA2 and age was as high as 64%^19^. Therefore, the association between Lp-PLA2 and CAD observed in elderly patients may be different in young Chinese patients. To our knowledge, our study was the first to show that Lp-PLA2 mass was associated with the risk of ACS after adjusting for conventional risk factors, including smoking, LDL-C, HDL-C, TG and hs-CRP. These results were supported by the CARDIA study, which showed that elevated Lp-PLA2 mass was associated with a high coronary artery calcium score in young Black and Caucasian adults^20^. Another study also showed that Lp-PLA2 levels were positively correlated with subclinical coronary atherosclerosis detected by coronary computed tomography angiography (CTA) in young patients^21^.

Oxidative stress and inflammation are important processes in the pathogenesis of atherosclerosis. Pathologically, Lp-PLA2 uses ox-LDL as a substrate and produces free fatty acids and lysophosphatidylcholine, a powerful pro-inflammatory factor, which subsequently results in endothelial dysfunction, foam cell formation, necrotic lipid-core expansion and fibrous cap thinning^22^. Thus, Lp-PLA2 has been used as a marker representing a link between inflammation, oxidized lipoproteins and plaque instability. For the first time, our study showed that Lp-PLA2 levels were positively correlated with ox-LDL in patients. These results were supported by a prior animal study that showed that in hypercholesterolaemic pigs, ox-LDL robustly increased Lp-PLA2 mRNA expression in inflammatory cells^23^. Our study found that the association between Lp-PLA2 and risk of ACS became insignificant when adjusting for ox-LDL levels, indicating that the link between Lp-PLA2 and ACS may be dependent on the levels of ox-LDL. Our study differed from the Bruneck study, which demonstrated that oxidized phospholipids/apolipoprotein B-100 levels can predict 10-year CVD events independently of traditional risk factors. Increasing Lp-PLA2 activity further amplified the risk of CVD mediated by oxidized phospholipids/apolipoprotein B-100^24^. The discrepancy between these studies may be caused by different ethnicities and ages. The Bruneck study included European subjects between 40 and 79 years old, while our study included Chinese patients less than 55 years of age.

The lack of clear improvement in risk prediction for ACS by Lp-PLA2 when adjusted for ox-LDL levels, suggests that Lp-PLA2 is a marker of oxidative stress rather than an independent risk factor for CVD. This assumption was supported by a recently published Mendelian randomization study and randomized clinical trials. A Japanese study showed that Lp-PLA2 activity was associated significantly with intima-media thickness and plaque in the carotid artery, however, Mendelian randomization did not support LpPLA2 as a causative factor^25^. The STABILITY (Stabilization of Atherosclerotic Plaque by Initiation of Darapladib Therapy)^26^ and the SOLID-TIMI 52 (Stabilization of Plaque Using Darapladib-Thrombolysis in Myocardial Infarction 52)^27^ studies evaluated the clinical efficacy and safety of the Lp-PLA2 inhibitor (darapladib) in patients with stable CAD and ACS, respectively. In both studies, darapladib did not reduce the risk of CVD or all-cause mortality compared with the placebo.

There were some potential limitations of this study. First, because of the case-control design of the study, we could not definitively evaluate the cause-effect associations, and reverse causality may exist. However, unlike hs-CRP, Lp-PLA2 mass does not behave as an acute-phase reactant. Lp-PLA2 levels were unaffected in ACS up to 84 h^28^. In our study, Lp-PLA2 levels were determined on the day after enrollment to limit the possibility of reverse causality. Second, Lp-PLA2 activity was not determined in our study. The role of Lp-PLA2 mass and activity on prediction of CVD may be different, although it was previously reported that there was a strong correlation between them^14^. Data from the JUPITER (Justification for the Use of Statins in Prevention: an Intervention Trial Evaluating Rosuvastatin) trial found that among those study participants allocated to placebo, Lp-PLA2 activity but not Lp-PLA2 mass were associated with incident cardiovascular events^29^. Similarly, the PROGRESSA (Metabolic Determinants of the Progression of Aortic Stenosis) study indicated that although there was no significant association between Lp-PLA2 activity or mass and aortic stenosis progression in the whole cohort, increased Lp-PLA2 activity was associated with a faster stenosis progression rate in the subset of patients with mild aortic stenosis^30^. Based on these results, we believe that although in present study cohort, Lp-PLA2 mass appears to be a marker of oxidative stress and inflammation rather than an independent risk factor for CVD, a possible role for LP-PLA2 activity and risk of ACS cannot be completely ruled out. Third, the present study included a small sample from a single center and the analysis may therefore be underpowered. We found that although there was a strong trend for a relationship between ACS and Lp-PLA2 after adjustment of ox-LDL (OR = 1.69, 95%CI = 0.90–3.17), this result did not reach statistical significance. Further studies with larger sample sizes are needed to explore the role of Lp-PLA2 in ACS.

In conclusion, the present study demonstrated that Lp-PLA2 concentration was positively associated with ox-LDL levels in young Chinese patients with ACS. The link between Lp-PLA2 and risk of ACS may be dependent on ox-LDL levels. These results support Lp-PLA2 as a marker of oxidative stress and inflammation rather than an independent risk factor for CVD.

## Methods

### Subjects

The inclusion criteria for patients with ACS included the following. (1) Young patients with age ≤55 years^31,32^. (2) First attack of chest discomfort within 2 months. (3) With ≥50% stenosis of the lumen diameter in ≥1 major coronary artery (including the left main coronary artery, left anterior descending branch, left circumflex branch and right coronary artery) quatified by CAG. CAG was performed using the standard Judkins technique through the femoral or radial artery with the Allura Xper FD20 (Philips, Amsterdam, Netherlands) and analysed by two independent interventional cardiologists.

Age-matched individuals with no obstructive coronary stenosis by CAG (no or less than 50% stenosis) were enrolled in the control group. It has been reported that a negative predictive value by coronary CTA for coronary artery stenosis is similar to that by CAG^33^. We also included 185 patients with negative findings by coronary CTA as controls. The final control group included 237 patients.

The reasons for performing CAG in the control group were because of chest pain with suspected ischemic findings, such as abnormal ST segment deviation in electrocardiograph, positive results for treadmill test, ischemic findings by myocardial perfusion scintigraphy, regional dyskinesia in echocardiography, or difficulty in differential diagnosis.

The exclusion criteria included the following. (1) Patients with suspected acute myocardial infarction (defined by chest pain and elevated troponin), while without obstructive coronary stenosis were excluded. These patients are with diverse pathophysiological mechanisms and different with those with obstructive CAD. (2) Patients with uncontrolled infectious diseases, autoimmune disease, severe renal dysfunction (creatinine ≥265 μmol/L), malignancy, pregnancy, hormone replacement therapy after menopause. These conditions may have significant influence on evaluations of disease markers. (3) Individuals with psychiatric disorders (including anxiety, depression or bipolar disorders) were also excluded due to potential effects on levels of ox-LDL^34^.

The study complied with the Declaration of Helsinki and was approved by the Ethics Committee of Shunde Hospital, Southern Medical University. Written informed consent was obtained from all participants.

### Laboratory measurements

Fasting venous blood samples were collected to measure glucose, TC. HDL-C, triglyceride and creatinine levels using the Olympus AU2700 Automatic Biochemical Analyzer (Japan). LDL-C was calculated using the Friedewald equation. Hs-CRP was determined by the high-sensitivity nephelometric method. Plasma ox-LDL was measured by sandwich enzyme-linked immunosorbent assay procedure (Mercodia, Uppsala, Sweden)^16^. The interassay coefficient of variation was 6.8%.

After centrifugation at 1500 × g for 10 min at 4 °C, plasma samples were stored at −80 °C for future measurements of Lp-PLA2 mass, as long-term stability of Lp-PLA2 has been demonstrated^35^. Briefly, Lp-PLA2 mass was measured using a commercial enzyme-linked immunosorbent assay kit (Tianjin Kangerke Bioscience, Tianjin, China). The interassay coefficient of variation was 4.8%. All measurements were performed according to the manufacturers’ instructions.

### Definition of risk factors for CVD

Conventional risk factors for CVD included the following. (1) Hypertension was defined according to Joint National Committee on Prevention, Detection, Evaluation, and Treatment of High Blood Pressure (JNC) VII guideline^36^. (2) Diabetes mellitus was defined based on American Diabetes Association criteria or as history of treatment with either insulin or oral hypoglycaemic medication^37^. (3) Dyslipidaemia was defined as TC ≥ 5.2 mmol/L, LDL-C ≥ 3.4 mmol/L, HDL-C < 1.03 mmol/L and/or TG ≥ 1.7 mmol/L according to the 2007 Guidelines for Prevention and Treatment of Dyslipidemia in Adults in China or history of receiving antidyslipidaemia agents^38^. (4) BMI was indicative of being overweight (24–27.9 kg/m^2^) or obese (≥28 kg/m^2^) according to Chinese criteria^39^. (5) For smoking, participants were classified as current smokers if they reported smoking regularly during the past 1 year or non-smokers (including former smokers if they had stopped smoking for at least 1 year and never smokers). (6) Positive family history of premature CVD was defined as a diagnosis of CVD in a first-degree male relative <55 years of age or first-degree female relative <65 years of age. (7) The eGFR was calculated using the modified Modification of Diet in Renal Disease equation adapted for Chinese subjects^40^.

We also calculated the absolute 10-year CVD events risk scores based on the Framingham risk score system modified by the National Cholesterol Education Program Adult Treatment Panel III (NCEP ATP III) guideline^41^, which include major independent risk factors, such as age, hypertension, dyslipidaemia, diabetes, family history of premature CVD and smoking status.

### Statistical analysis

Statistical analysis was performed using the Statistical Package for Social Science software version 22.0 (SPSS Inc., Chicago, IL, USA). Categorical variables are expressed as percentages. Continuous variables are presented as median (inter-quartile range) or mean (standard deviation) as appropriate.

After testing for normality using the Kolmogorov–Smirnov test, continuous variables were compared using the Mann–Whitney *U* or Student *t*-test, and categorical variables were compared with the chi-square or Fisher’s exact test as appropriate. The Pearson correlation for normal variables or Spearman correlation for skewed variables was used to evaluate the associations between study parameters. Multiple logistic regression analysis was performed to evaluate the risk factors for CVD. Variables such as age, smoking history, hypertension, DM, family history of CVD, BMI and TG, TC, HDL-C, LDL-C, hs-CRP, eGFR, ox-LDL and Lp-PLA2 levels were set as independent variables. Odds ratios (ORs) and 95% confidence intervals (CIs) were calculated. Multicollinearity (strong correlations among independent variables) was examined by collinearity diagnostic statistics. Variance inflation factor values >2.5 or tolerance <0.4 may indicate concern for multicollinearity in logistic regression models. *P* value < 0.05 was considered statistically significant.

### Data Availability

The dataset analyzed during the current study is available from the corresponding authors on reasonable request.

## References

[1] Levine GN (2016). ACC/AHA guideline focused update on duration of dual antiplatelet therapy in patients with coronary artery disease: A report of the American College of Cardiology/American Heart Association Task Force on Clinical Practice Guidelines. J Am Coll Cardiol..

[2] Li J (2015). ST-segment elevation myocardial infarction in China from 2001 to 2011 (the China PEACE-Retrospective Acute Myocardial Infarction Study): a retrospective analysis of hospital data. Lancet..

[3] Shah N, Kelly AM, Cox N, Wong C, Soon K (2016). Myocardial Infarction in the “Young”: risk Factors, presentation, management and prognosis. Heart Lung Circ..

[4] Rosengren A (2006). Age, clinical presentation, and outcome of acute coronary syndromes in the Euroheart acute coronary syndrome survey. Eur Heart J..

[5] Wang X (2017). Trend in young coronary artery disease in China from 2010 to 2014: a retrospective study of young patients ≤45. BMC Cardiovasc Disord..

[6] Matsis K (2017). Differing clinical characteristics between young and older patients presenting with myocardial infarction. Heart Lung Circ..

[7] Essilfie G (2016). Association of elevated triglycerides and acute myocardial infarction in young Hispanics. Cardiovasc Revasc Med..

[8] Ghatge M, Sharma A, Vangala RK (2016). Association of gamma-glutamyl transferase with premature coronary artery disease. Biomed Rep..

[9] Wu Y (2013). Hyperhomocysteinemia is an independent risk factor in young patients with coronary artery disease in southern China. Herz..

[10] Zhang Y (2016). HDL subfractions and very early CAD: novel findings from untreated patients in a Chinese cohort. Sci Rep..

[11] Tatli E, Ozcelik F, Aktoz M (2009). Plasma fibrinogen level may predict critical coronary artery stenosis in young adults with myocardial infarction. Cardiol J..

[12] Qi Y (2016). A previously unreported impact of a PLA2G7 gene polymorphism on the plasma levels of lipoprotein-associated phospholipase A2 activity and mass. Sci Rep..

[13] Li D (2017). Lipoprotein-associated phospholipase A2 in coronary heart disease: Review and meta-analysis. Clin Chim Acta..

[14] Thompson A (2010). Lipoprotein-associated phospholipase A(2) and risk of coronary disease, stroke, and mortality: collaborative analysis of 32 prospective studies. Lancet..

[15] Cai A (2015). Increased serum level of Lp-PLA2 is independently associated with the severity of coronary artery diseases: a cross-sectional study of Chinese population. BMC Cardiovasc Disord..

[16] Huang Y (2011). Plasma oxidized low-density lipoprotein is an independent risk factor in young patients with coronary artery disease. Dis Markers..

[17] Vittos O, Toana B, Vittos A, Moldoveanu E (2012). Lipoprotein-associated phospholipase A2 (Lp-PLA2): a review of its role and significance as a cardiovascular biomarker. Biomarkers..

[18] Garg PK (2015). Lipoprotein-associated phospholipase A2 and risk of incident cardiovascular disease in a multi-ethnic cohort: The multi ethnic study of atherosclerosis. Atherosclerosis..

[19] Ge PC (2016). Synergistic Effect of Lipoprotein-Associated Phospholipase A2 with Classical Risk Factors on Coronary Heart Disease: A Multi-Ethnic Study in China. Cell Physiol Biochem..

[20] Iribarren C (2005). Association of lipoprotein-associated phospholipase A2 mass and activity with calcified coronary plaque in young adults: the CARDIA study. Arterioscler Thromb Vasc Biol..

[21] Celik O (2015). Evaluation of lipoprotein-associated phosholipase A2 and plaque burden/composition in young adults. Coron Artery Dis..

[22] Rosenson RS, Hurt-Camejo E (2012). Phospholipase A2 enzymes and the risk of atherosclerosis. Eur Heart J..

[23] De Keyzer D (2009). Increased PAFAH and oxidized lipids are associated with inflammation and atherosclerosis in hypercholesterolemic pigs. Arterioscler Thromb Vasc Biol..

[24] Kiechl S (2007). Oxidized phospholipids, lipoprotein(a), lipoprotein-associated phospholipase A2 activity, and 10-year cardiovascular outcomes: prospective results from the Bruneck study. Arterioscler Thromb Vasc Biol..

[25] Ueshima H (2016). Lipoprotein-associated phospholipase A2 is related to risk of subclinical atherosclerosis but is not supported by Mendelian randomization analysis in a general Japanese population. Atherosclerosis..

[26] White HD (2014). Darapladib for preventing ischemic events in stable coronary heart disease. N Engl J Med..

[27] O’Donoghue ML (2014). Effect of darapladib on major coronary events after an acute coronary syndrome: the SOLID-TIMI 52 randomized clinical trial. JAMA..

[28] James SK (2005). An acute inflammatory reaction induced by myocardial damage is superimposed on a chronic inflammation in unstable coronary artery disease. Am Heart J..

[29] Ridker PM, MacFadyen JG, Wolfert RL, Koenig W (2012). Relationship of lipoprotein-associated phospholipase A(2) mass and activity with incident vascular events among primary prevention patients allocated to placebo or to statin therapy: an analysis from the JUPITER trial. Clin Chem..

[30] Capoulade R (2015). Impact of plasma Lp-PLA2 activity on the progression of aortic stenosis: the PROGRESSA study. JACC Cardiovasc Imaging..

[31] Pelletier R (2016). Sex Versus Gender-Related Characteristics: Which Predicts Outcome After Acute Coronary Syndrome in the Young?. J Am Coll Cardiol..

[32] Yang Y (2015). Perceived stress status and sympathetic nervous system activation in young male patients with coronary artery disease in China. Eur J Intern Med..

[33] Bittencourt MS, Hulten EA, Veeranna V, Blankstein R (2016). Coronary Computed Tomography Angiography in the Evaluation of Chest Pain of Suspected Cardiac Origin. Circulation..

[34] Maes M (2010). Increased plasma peroxides and serum oxidized low density lipoprotein antibodies in major depression: markers that further explain the higher incidence of neurodegeneration and coronary artery disease. J Affect Disord..

[35] Charniot JC (2013). Interpretation of lipoprotein-associated phospholipase A2 levels is influenced by cardiac disease, comorbidities, extension of atherosclerosis and treatments. Int J Cardiol..

[36] Chobanian AV (2003). Seventh report of the Joint National Committee on Prevention, Detection, Evaluation, and Treatment of High Blood Pressure. Hypertension..

[37] Expert Committee on the Diagnosis and Classification of Diabetes Mellitus. Report of the expert committee on the diagnosis and classification of diabetes mellitus. *Diabetes Care*. **26** Suppl 1, S5–S20 (2003). 10.2337/diacare.26.2007.s5 12502614

[38] Joint Committee for Developing Chinese guidelines on Prevention and Treatment of Dyslipidemia in Adults. [Chinese guidelines on prevention and treatment of dyslipidemia in adults]. *Zhonghua Xin Xue Guan Bing Za Zhi*. **35**, 390–419 (2007). 17711682

[39] Cui H (2011). Association factors of target organ damage: analysis of 17,682 elderly hypertensive patients in China. Chin Med J (Engl)..

[40] Ma YC (2006). Modified glomerular filtration rate estimating equation for Chinese patients with chronic kidney disease. J Am Soc Nephrol..

[41] Talbert RL (2002). New therapeutic options in the National Cholesterol Education Program Adult Treatment Panel III. Am J Manag Care..

